# Impact of graphitic carbon nitrides synthesized from different precursors on Schottky junction characteristics

**DOI:** 10.3906/kim-2012-45

**Published:** 2021-08-27

**Authors:** Orhan ALTAN

**Affiliations:** 1 Department of Chemistry, Vocational School of Technical Sciences, Mersin University, Mersin Turkey; 2 Department of Nano Technology and Advanced Materials, Institute of Science, Mersin University, Mersin Turkey

**Keywords:** Graphitic carbon nitride, Palladium nanoparticles, alloy nanoparticles photocatalyst, formic acid, dehydrogenation

## Abstract

Graphitic carbon nitride (g-CN) has gained wide interest in many areas, such as energy and the environmental remediation as a layered polymeric semiconductor that allows the formation of catalytically active Schottky junctions due to its proper electronic band structure. Interestingly, although it is known that the precursors used in the synthesis, can influence the properties of the g-CN, no detailed study on these effects on Schottky junctions could be found in the literature. In this research, the effects of g-CNs synthesized by thermal polycondensation of different precursors on the photocatalytic efficiency of Schottky junctions were investigated. For this purpose, urea, thiourea, melamine, and guanidine hydrochloride were used as different precursors, while the photocatalytic dehydrogenation of formic acid was used as a test reaction. The Schottky junctions were formed by decorating the as-prepared g-CNs with AgPd alloy nanoparticles (NP), which were synthesized by reduction of Ag and Pd salts with NaBH_4_. The structural, electronic and charge carrier dynamics of all prepared structures have been fully characterized by TEM, XRD, BET, XPS, UV-Vis DRS, PL, and PL life measurements. The results showed that the charge transfer dynamics of g-CNs surface defects are more effective in the photocatalytic performance of Schottky junctions than in structural features such as the size of the metal NPs or the surface area of the catalysts.

## 1. Introduction

**Scheme 1:**
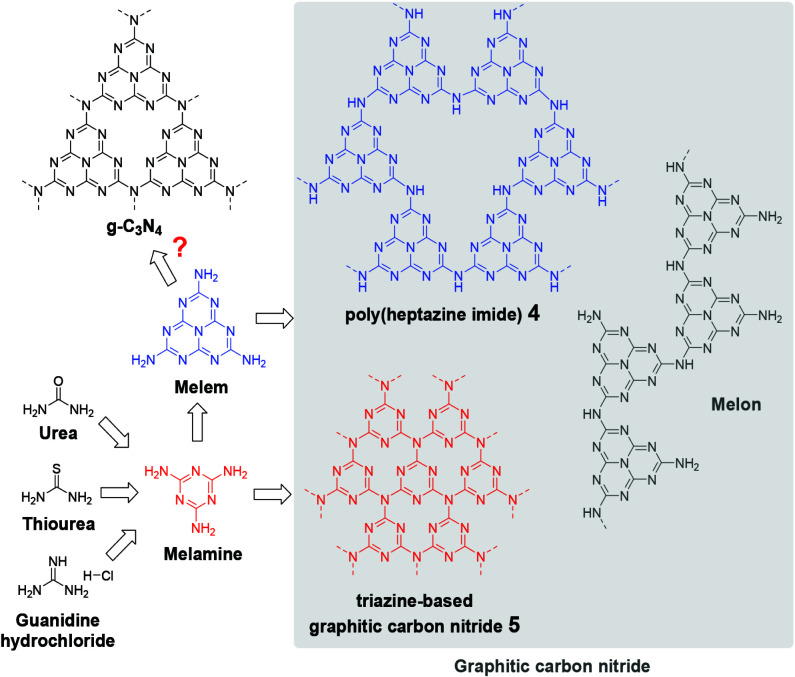
Schematic representation of the graphitic carbon nitride formation.

Photocatalysis being able to transform solar energy into chemical energy in order to control the chemical reactions is regarded as a very promising approach [1–4]. Therefore, the development of photocatalysts that are effective under visible light is a very important task. So far, the most studied visible light active semiconductors are those formed by transition metal ions with counter-anions such as chalcogens [5,6]. The efficacy of these traditional inorganic semiconductors is constrained in terms of the nature of their active sites, hence the number of applications they can perform [7]. On the other hand, graphitic carbon nitride (g-CN) stands out as a remarkable polymeric semiconductor with its unique properties such as high thermal, chemical and physical stability along with the appropriate band structure [8], while organic polymeric catalysts have significant stability issues [9]. The g-CN can be synthesized by several methods including solvothermal [10], chemical vapor deposition (CVD) [11], and plasma sputtering reaction deposition [12], whereas the thermal polycondensation method is the most attractive one due to its simplicity and low cost [8]. Until today, the “perfect” graphitic carbon nitride (g-C_3_N_4_) material, consisting only of C and N bonds in the form of p-conjugated flat poly(tri-s-triazine) sheets interconnected through tertiary amines, has not been synthesized [13–15]. The poly(heptazine imide) 4 and triazine-based graphitic carbon nitride 5 were the closest to g-C_3_N_4_ synthesized to date (Scheme 1) [13,16]. However, in most of the published papers, carbon nitrides have been described as g-C_3_N_4_, but essentially, it would be more appropriate not to provide a detailed structure formulation due to limited knowledge of the actual structure of the materials obtained. Thus, in this study, it was preferred to use g-CN to describe carbon nitrides. 

Understanding the carbon nitride as a polydisperse and a polycrystalline polymer allows the investigation of a number of synthetic techniques that will be used to produce a variety of morphological and chemical properties [13]. For instance, alteration of procedures or precursors in the synthesis may result in g-CNs having different electronic and morphological properties [17]. To this end, a number of attempts have been made to compare the effect of synthesis procedures on the properties of g-CNs obtained from various precursors including melamine [18], urea [19], thiourea [20], cyanamide [21], and dicyandiamide [22]. In fact, this approach served as a novel guideline for advanced photocatalysis with notable improvements in photocatalytic activities rather than the search for the perfect single crystal. However, practical applications still have some problems due to the various obstacles and shortcomings of pristine g-CN. These problems can be listed as; high recombination rate of charge carriers, low charge mobility, and the inability of pristine g-CN to absorb light with a wavelength more than 460 nm [23]. In order to overcome these drawbacks, junctions can be formed by combining g-CN with a second material so that the photocatalytic efficiency can be improved by facilitating the separation of the charge carriers [24]. This second material can be a conductor forming a Schottky junction or a semiconductor. In photocatalysis, transition metal nanoparticles (NPs), also serve as co-catalysts, and are often used as conductors for the formation of Schottky junctions [15,24]. A number of studies have separately reported the photocatalytic activities of Schottky junctions formed by various combinations of g-CNs with metal NPs [15]. Interestingly; however, no studies were found examining the impact of precursors or synthesis method of g-CNs on the characteristics of Schottky junctions generated from g-CNs, in contrast to the literature based on the bare g-CN [23].

**Scheme 2:**
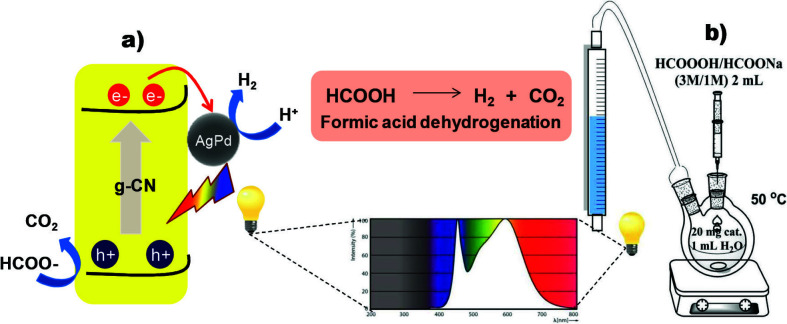
Representation of photocatalytic formic acid dehydrogenation a) mechanism of the reaction b) experimental setup.

In this study, it was aimed to investigate the effect of g-CNs synthesized by the polycondensation of the most common precursors, such as melamine (M), urea(U), thiourea (T), and guanidine hydrochloride (G), on the photocatalytic activity of Schottky junctions. For this purpose, photocatalytic dehydrogenation of formic acid was chosen as a model reaction due to the relatively ease of monitoring the progress of the reaction, in addition to its importance [25]. It should be noted that the mechanism of photocatalytic formic acid dehydrogenation which emphasized the importance of photogenerated holes in the valence band of the semiconductor, is different from heterogeneous catalysis. Such that, formate ion is oxidized to CO_2_ on the photogenerated holes, which is the rate-limiting step in H_2_-evolution [26–28]. Thus, the charge separation ability of the photocatalyst would be essential for the reaction rate. (Scheme 2). Moreover, previous studies have reported that the best photocatalytic activity for the dehydrogenation of formic acid has been observed in AgPd alloy NPs, in particular, the ones in the 1:9 (Ag:Pd) ratio [29–31]. Therefore, the Schottky junctions, g-CN(X)-AgPd (X: M, U, T, G), were prepared by the reduction of the corresponding aqueous solutions of metal salts on g-CNs with NaBH_4_. Then the photocatalytic activities of Schottky junctions on the dehydrogenation of formic acid reaction were examined and discussed in detail.

## 2. Experimental

### 2.1. Materials

Urea (CH_4_N_2_O) (99%), thiourea (CH_4_N_2_S) (99%) melamine (C_3_H_6_N_6_) (98%) guanidine hydrochloride (CH_5_N_3_·HCl) sodium borohydride (NaBH_4_) (>96%) (Merck), silver nitrate (AgNO_3_) (99.9%) potassium tetrachloropalladate(II) (K_2_PdCl_4_) (99.99% metals basis) (Alfa Aesar), formic acid (HCOOH) (>98%), and sodium formate (HCOONa) (≥99%) (Sigma-Aldrich) were purchased from various companies and used as received without further purification.

### 2.2. Instrumentation

The diffraction patterns of photocatalysts were recorded on the powder X-ray diffraction (Bruker D8) employing Cu Kα radiation. Transmission electron microscopy (TEM; FEI- TALOS F200S TEM 200 kV) was used for analyzing the morphology and particle size of the samples. UV−visible diffuse-reflectance (UV−Vis DRS) spectra were recorded by a UV−vis spectrophotometer (Shimadzu UV-3600 UV-Vis-NIR Spectrophotometer) with BaSO_4_ as the reference. X-ray photoelectron spectroscopy (XPS, Thermo K-Alpha) was used for determining the chemical states and surface compositions of the photocatalysts. The binding energy correction was performed by taking the C1s reference peak of carbon atom at 284.5 eV. The photoluminescence (PL) spectra and time-resolved photoluminescence (TRPL) spectra of the photocatalysts excited at 325 nm were recorded on Agilent Cary Eclipse PL spectrophotometer and Edinburgh Instruments FLS1000 Spectrometer, respectively. Inductively coupled plasma–mass spectroscopy (ICP–MS) measurements were carried out on Agilent 7700x ICP mass spectrometer equipped with ASX-500 auto sampler. ICP-MS sample preparation was done by using StartD Milestone microwave digestion.

### 2.3. Synthesis of g-CNs

The g-CNs were synthesized from the precursors such as urea, thiourea, melamine, and guanidine hydrochloride, by using a hard template-free thermal polycondensation method [32]. The typical procedure involves heating 10 g of the selected precursor which was grounded and maintained at 80 °C for 1 h in a crucible with a cover to 550 °C for 4 h in a muffle furnace. The resulted yellowish powder was collected for use without further treatment

### 2.4. Synthesis of Schottky junction photocatalysts

g-CN-AgPd nanocomposites were synthesized by direct precipitation method [29,31]. Typically, 100 mg of as-prepared g-CN was dispersed in 10 mL of an aqueous solution of AgNO_3_ (0.02 mmol) and K_2_PdCl_4_ (0.18 mmol) added and the resultant mixture was stirred overnight at room temperature. Next, a fresh NaBH_4_ aqueous solution (1 M, 2 mL) was added dropwise into the mixture in an ice-bath. This mixture was stirred at room temperature for further 3 h and then the solid was collected by centrifuges, washed with water and ethanol twice. Finally, the resultant solid was dried at 80 °C for 3 h.

### 2.5. Photocatalytic activity of as-prepared Schottky junctions

Twenty mg of the catalyst and 1 mL of distilled water were put in a jacketed-glass reactor with a gas outlet and sealed with a septum in a typical catalytic activity measurement. In a home-designed cubic photoreactor cabin that has four LED (5W) lamps on each vertical side, the reactor was illuminated. FA/SF mixture (1.5M/0.5M, 2 mL) was added by syringe at 323 K. The generated gas was monitored using the gas burette system.

## 3. Results and discussion

A series of graphitic carbon nitrides were synthesized from the most commonly used precursors in literature, which are melamine, urea, thiourea, and guanidine hydrochloride, using the same thermal polycondensation method [32]. It is known that prolonged reaction time causes thermal oxidation process which resulted exfoliation of the bulk carbon nitrides into smaller sheets (thermal exfoliation) [33,34]. Therefore, g-CNs were synthesized by the method used for the thermally exfoliated g-CN with the highest surface area, ever [32]. Schottky junctions were prepared by AgPd alloy NPs formed by the reduction of absorbed metal salts (AgNO_3_ and K_2_PdCl_4_) by an aqueous NaBH_4_ solution on the as-prepared g-CNs [29,31].

**Figure 1:**
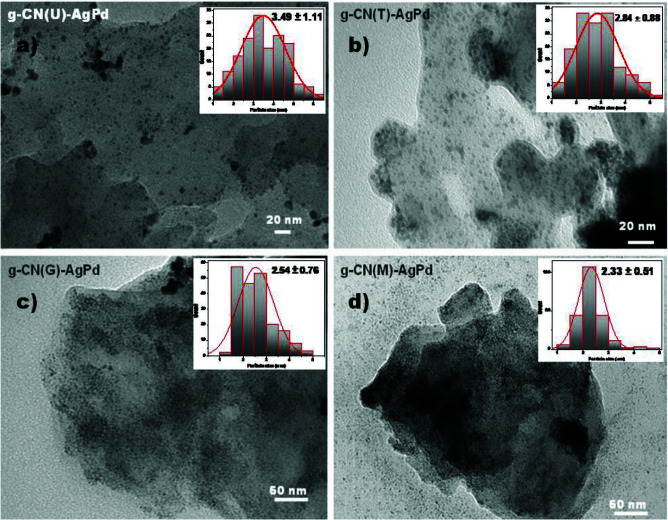
TEM image of a) g-CN(U)-AgPd, b) g-CN(T)-AgPd, c) g-CN(G)-AgPd, d) g-CN(M)-AgPd nanocomposites. Insets of figures are representing the graphs of size distribution of AgPd alloy NPs.

Figure 1 shows the transmission electron microscopy (TEM) images of prepared Schottky junctions. TEM images indicate that g-CN(U) has a thin-layer structure (increased relative light transmittance) and a more porous surface morphology compared to other structures (Figure 1a). It has been observed that the AgPd alloy NPs formed on g-CN(U) are the largest particles among the four Schottky junctions along with the widest particle size distribution (3.49 ± 1.11 nm). On the other hand, an almost similar particle size and distribution were observed in g-CN(T) (2.84 ± 0.88 nm) (Figure 1b) and g-CN(G) (2.54 ± 0.76) (Figure 1c), while the smallest particle size and distribution was found in g-CN (M) (2.33 ± 0.51) (Figure 1d). Furthermore, the light gray portion of the TEM image of g-CN(M)-AgPd (Figure 1d) decorated with AgPd NPs indicates that g-CN(M) has larger layers than the others. The same image also shows a smoother surface and a bulkier structure. This observation is also in agreement with the results obtained from the N_2_ adsorption-desorption isotherms (vide infra). On the other hand, it is known that the pores are created chemically active surface defect sites thus these sites could be an attraction center for many structures [35] including metal atoms. Thus, it can be argued that this variability in the sizes of the AgPd NPs is related to the morphology of the materials. For instance, the smoothest surface (g-CN(M)) has the smallest NPs while the most porous surface (g-CN(U)) has the largest NPs. Similarly, on g-CN(T)-AgPd with larger pores relative to g-CN(G)-AgPd, slightly larger NPs were observed. It should be noted that Ag and Pd contents of the Schottky junctions are determined by ICP-MS analysis. Pd contents of g-CN(U)-AgPd, g-CN(T)-AgPd, g-CN(G)-AgPd, and g-CN(M)-AgPd were determined as 15.9, 15.5, 15.7, and 15.0%, respectively. Furthermore, Ag: Pd ratios were found as 14:86, 15:85, 14:86, and 13:87 for g-CN(U)-AgPd, g-CN(T)-AgPd, g-CN(G)-AgPd and g-CN(M)-AgPd, respectively.

**Figure 2:**
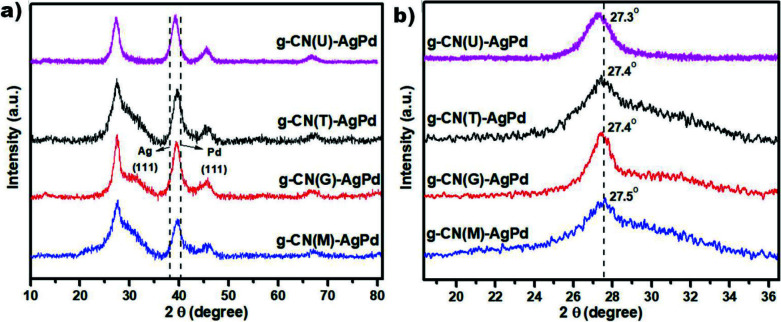
XRD patterns of a) synthesized Schottky junctions, b) 002 diffraction plane of g-CNs.

Figure 2a illustrates the XRD patterns of g-CN(U)-AgPd, g-CN(T)-AgPd, g-CN(G)-AgPd, and g-CN(M)-AgPd. The diffraction peaks at 2q = 13.1° and around 27.5° are attributed to (100) and (002) diffraction planes of g-CN, respectively. Moreover, a closer look at the peaks of (002) diffraction planes revealed slight shifts to low angles for g-CN(U)-AgPd, g-CN(T)-AgPd and g-CN(G)-AgPd structures found at 27.3°, 27.4°, and 27.4°, respectively (Figure 2b). The shifts to these lower angles correspond to an increase in the spacing of the interlayer which may be due to the prolonged pyrolysis time causing the separation and buckling of the nanosheets, thus making the materials more amorphous [34]. In other words, it can be stated that g-CN(M) has the most crystalline bulk structure while g-CN(U) has the least crystalline and exfoliated structure. The peaks at 39.5° correspond to the AgPd alloy NPs which have a slight shift to Ag (111) plane (at 2q = 38.7°) [36] compared to the Pd(111) diffraction plane (at 2q = 40.1°) [37] supports the existence of AgPd alloy structure in all nanocomposites [31,38].

**Figure 3:**
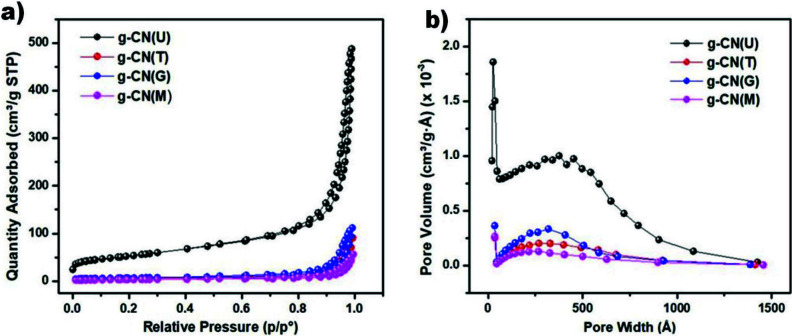
a) The nitrogen adsorption/desorption isotherms, b) the corresponding pore-size distribution curves of the prepared structures.

Figure 3 displays the N_2_ adsorption-desorption isotherms and Barrett–Joyner–Halenda (BJH) pore-size distribution curves of g-CN(U), g-CN(T), g-CN(G), and g-CN(M). The adsorption-desorption isotherms in Figure 3a reveal that all samples are type IV, suggesting the presence of mesopores (2–50 nm). Furthermore, the pore size distribution of the materials (Figure 3 b) supported the formation of mesopores. In all samples, small (2–5 nm) and large (5–50 nm) mesopores and macropores (50–80 nm) can be observed. The hysteresis loops are of type H3, which suggests that the aggregates of plate-like particles contain slit-shaped pores. Textural parameters of synthesized g-CNs are shown in Table 1. The results indicate that g-CN(U) has exfoliated into thin sheets and split into small layers during the thermal process. Unlike others, g-CN (G) has also followed the exfoliation process, although not as much as g-CN(U). It can be claimed that the random aggregation of these layers resulted in the formation of abundant mesopores [39]. These mesopores constituted the majority of the pore volume of the structures (Table 1). In contrast, the presence of large pores on the g-CN(U) and g-CN(T) surfaces were demonstrated by TEM images, as mentioned above. The polycrystalline structure of the graphitic carbon nitrides can be an explanation for the various responses of g-CNs, obtained from different precursors, to the thermal exfoliation process. Although poly-heptazine is energetically favored, graphitic carbon nitrides, as shown in Scheme 1, consist of two main structures, poly (heptazine imide) 4 and triazine-based graphitic carbon nitride 5 (Scheme 1) [13,15]. It can be envisaged that poly-heptazine imide units can create stronger interactions via hydrogen bonds than triazine based ones. Therefore, the strong interactions between g-CN(M) layers can be attributed to the poly-heptazine imide richness of the g-CN(M).

**Table 1:** Textural parameters of prepared Schottky junctions.

Structure	BET surface area(m²/g)	Average surface area of pores (m²/g)	Average pore volume (cm³/g)	Average particle size (nm)
g-CN(U)	183.04	140.26	0.74	32.78
g-CN(G)	22.16	21.78	0.17	270.76
g-CN(T)	18.73	15.95	0.14	320.40
g-CN(M)	11.11	10.43	0.09	540.29

**Figure 4:**
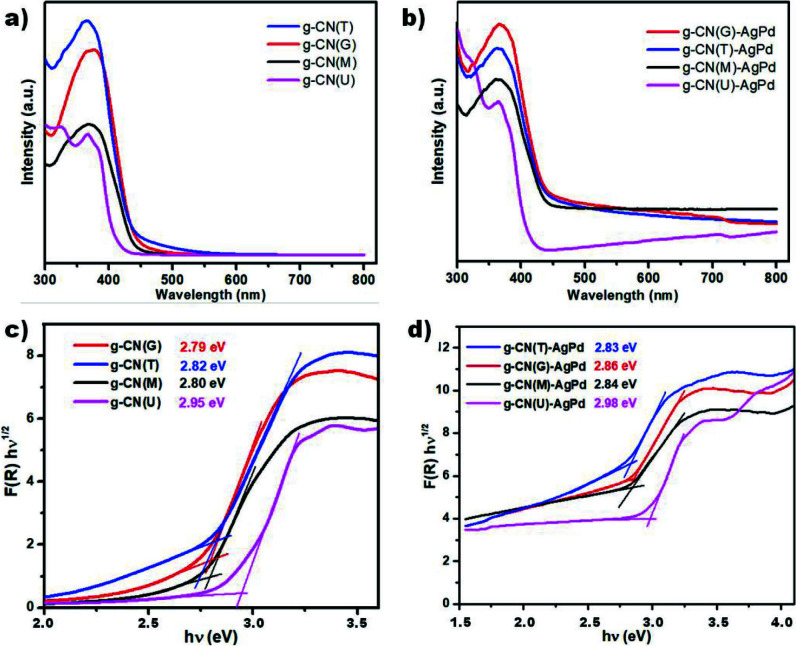
UV-Vis DRS absorption spectra and Tauc plots of prepared structures; a, c) g-CN and b, d) g-CN-AgPd.

UV-Vis DRS spectra of the pristine g-CNs and the corresponding Schottky junctions were given in Figure 4a and 4b **,** respectively. The bandgap energies of both pristine g-CN and Schottky junctions were evaluated by the following formula:

αhν = A(hν – E_gap_)^n/2^

where A is a constant, h is the Planck constant, ν is the light frequency, α is the absorption coefficient, n is 1 (direct band gap), and E_gap_ is the bandgap energy. As expected [8], it is observed that all graphitic carbon nitrides have band gaps of around 2.8 eV, except for g-CN(U) (2.95 eV). This unexpected bandgap observed in g-CN(U) can be attributed to the quantum size effect when the thin layered (nanosized) structure was formed [40,41]. No major changes in band gaps were observed after the inclusion of AgPd alloy NPs into the structures, but an increase in light absorption capabilities was detected, possibly due to the defect sites caused by metal NPs [42].

**Figure 5:**
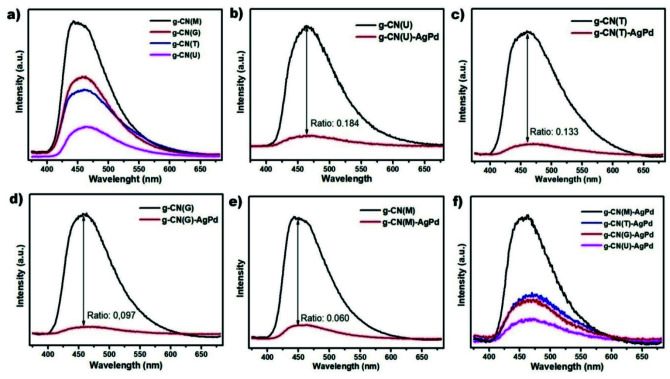
PL spectra comparison of; all g-CN structures (a), g-CN(U)/g-CN(U)-AgPd (b), g-CN(T)/g-CN(T)-AgPd (c), g-CN(G)/g-CN(G)-AgPd (d), g-CN(M)/g-CN(M)-AgPd (e), and all g-CN-AgPd structures (f).

The steady-state photoluminescence (PL) spectra can provide valuable information on the radiative recombination that occurs in semiconductor materials. The results, shown in Figure 5a, reveal that g-CN(M) has the highest radiative recombination rate, whereas g-CN(U) has the lowest. These findings are expected from a thin layer of g-CN(U) decorated with surface defects and a bulk g-CN(M) with relatively high crystallinity and are consistent with the results of the literature [32,40,43]. The inclusion of the AgPd alloy NPs into the structure was resulted in decreasing the radiative recombination in all nanocomposites (Figure 5b-e). However, the contribution of AgPd alloy NPs to the reduction of radiative recombination varies. The ratio of PL peak intensity of pristine g-CN to the Schottky junction peak intensity can provide information on the contribution of AgPd alloy NPs to the reduction of radiative recombination. The ratio of PL intensities of pristine g-CN to Schottky junctions was calculated as 0.184, 0.133, 0.097, and 0.060 for g-CN(U), g-CN(T), g-CN(G), and g-CN(M) respectively. Although the PL peak intensity of g-CN(M) is still too high (Figure 5f) it can be inferred that the Schottky junction formation is most effective in suppressing the destructive recombination in the g-CN(M) compared to others. It can therefore be argued that the defect sites created by AgPd NPs have a much more pronounced effect on facilitating the separation of charges. This argument is also in agreement with the results of TEM, BET and XRD analyzes, which reveal a more crystalline structure and a smooth surface morphology, of g-CN(M). Figure 5f shows the comparative PL emission spectra of all Schottky junctions, indicating that g-CN(U)-AgPd has the lowest radiative recombination, while g-CN(M)-AgPd has the highest. It is known that pores generate surface defects that serve as trapping sites for photo-induced charge carriers to suppress the recombination of electrons and holes [35]. Similarly, metal NPs can establish surface defect sites at contact points as they come together with a semiconductor. Thus, the findings are in agreement with the surface morphology of the samples examined.

**Figure 6:**
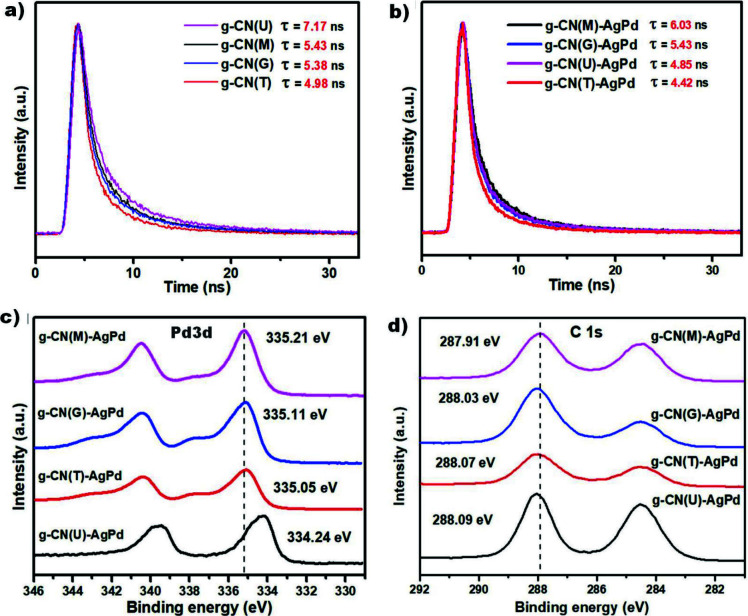
TRPL spectra of a) g-CN and b) g-CN-AgPd structures. Comparison of c) Pd 3d, and d) C 1s core-level XPS spectra of synthesized g-CN-AgPd structures.

The time-resolved fluorescence decay spectra of samples which gave the lifetime of charge carriers by fitting the decay curves, provides useful information about the charge-transfer dynamics [44]. Figure 6a illustrates the time-resolved fluorescence decay spectra for g-CNs obtained from different precursors. The long lifetime of the charge carriers indicates the efficiency of charge separation process. When the lifetime of charge carriers is considered together with the PL intensities, it can be argued that the most efficient charge separation process occurred in the g-CN(U). On the other hand, the observation of relatively long lifetime of the charge carriers in g-CN(M) can be due to the bulk structure of g-CN(M), which causes charge carriers to reach the surface in a much longer time [39,45]. After the insertion of AgPd alloy NPs into the structures, it was found that lifetimes were shortened in structures with pores on their surfaces, whereas lifetimes were extended in smoother structures (Figure 6b). In general, due to their contribution to the charge separation process, Schottky junctions are expected to lead to prolonged lifetimes, while shortened lifetimes can also be observed in thin layered structures with surface defects due to the existence of paths that allow charges to reach the surface more easily [45,46]. The faster access of the charge carriers to the surface also means that the metal is easier to enrich with electrons. In this context, the greatest change in lifetime was observed in the g-CN(U)-AgPd, and this finding suggests that metal NPs are better enriched with electrons in g-CN(U)-AgPd. These types of electron flows were supported by the Pd3d core-level X-ray photoelectron spectra (XPS) of the related samples (Figure 6c). The electron density of Pd atoms is highest in g-CN(U)-AgPd and lowest in g-CN(M)-AgPd considering that the binding energy is inversely related to the electron density. A reverse trend that implies the similar correlation was also observed in C1s core-level XPS of the same samples (Figure 6d).

**Figure 7:**
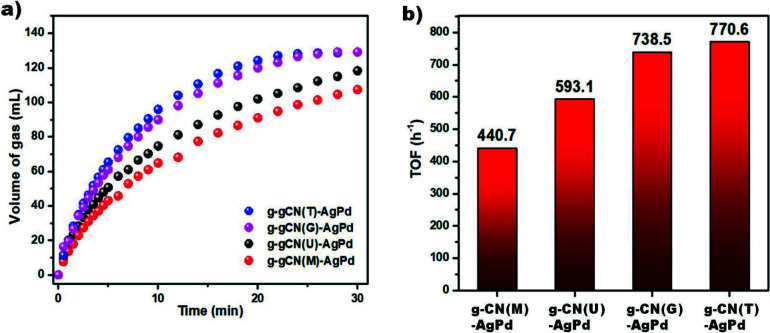
a) Time versus volume of gas generation in the presence of synthesized Schottky junctions under visible light illumination at 50  C, b) The TOFs of synthesized Schottky junctions. (The reported TOFs are the initial TOFs which were calculated according to the following equation when 20% of the total expected gas released: [TOF = mol H2/(mol Pd   h)]. The mole of Pd was determined by ICP-MS analysis).

The photocatalytic performances of prepared Schottky junctions were investigated through dehydrogenation of formic acid (Figure 7a). Although g-CN(M)-AgPd carried the smallest NPs on it, compared to the others, it displayed the worst photocatalytic activity (Figure 7b). This result may be referred to the low surface area of g-CN(M)-AgPd. However, the photocatalytic performance of g-CN(U)-AgPd, which has approximately 10 times more surface area than others, is only slightly higher than g-CN(M)-AgPd. It should therefore be considered that the charge carrier dynamics may also be effective on the photocatalytic performance of the examined structures. For the ease of review, all results obtained from structural, electronic and charge dynamics analyses are summarized in Table 2.

**Table 2:** Summary of the characterization data for the synthesized structures.

Catalyst	Surface area* (m2/g)	Pore volume * (cm³/g)	Average NP size (nm)	Pd content (wt%)	Pd3d BE (eV)	Egap (eV)	PL intens. ratio	PL lifetime(ns)
g-CN(U)-AgPd	183.04	0.74	3.49 ± 1.11	15.9	334.24	2.98	0.184	4.85
g-CN(T)-AgPd	18.73	0.14	2.84 ± 0.88	15.5	335.11	2.83	0.133	4.42
g-CN(G)-AgPd	22.16	0.17	2.54 ± 0.76	15.7	335.05	2.86	0.097	5.43
g-CN(M)-AgPd	11.11	0.09	2.33 ± 0.51	15.0	335.21	2.84	0.060	6.03
*For g-CNs								

According to the photocatalytic formic acid dehydrogenation mechanism, formate ion oxidizes to CO_2_ on the photogenerated holes, while H^+^ ion reduces to H_2_ on the metal NPs (Scheme 2)[28]. Therefore, in order to reach the effective photocatalysts, metal NPs should be enriched by electrons, at the same time, the stability of photogenerated holes located on the semiconductor surface should be improved. PL, TRPL, and XPS analyses (Table 2) revealed that the charge separation ability and charge carrier mobility is the best in g-CN(U)-AgPd which followed by g-CN(T)-AgPd and g-CN(G)-AgPd. Good charge separation ability creates more stable photogenerated holes while high charge carrier mobility results in enriched metal NPs with electrons. Thus, it could be expected that the g-CN(U)-AgPd should be the best photocatalyst which followed by g-CN(T)-AgPd and g-CN(G)-AgPd. However, the photocatalytic activity tests although revealing the high performance of g-CN(T)-AgPd and g-CN(G)-AgPd, the activity of g-CN(U)-AgPd has lagged behind. While the g-CN(U)-AgPd is the most efficient structure in terms of charge carrier dynamics, the light absorption ability of g-CN(U)-AgPd, which is crucial for the entire process, is limited. Actually, even though the charge carrier dynamics is very important for the photocatalytic processes; it is not the only factor that affects the photocatalytic activity. The adsorption of substrates to the catalyst surface is also important for the occurrence of photocatalytic redox reactions on the surface. It is known that surface defects can improve the substrate catalyst interactions which results in the enhanced catalytic activity. Thus, owing to the porous nature of g-CN(T), the surface defects of g-CN(T)-AgPd may be the reason for higher photocatalytic activity relative to g-CN(G)-AgPd.

**Figure 8:**
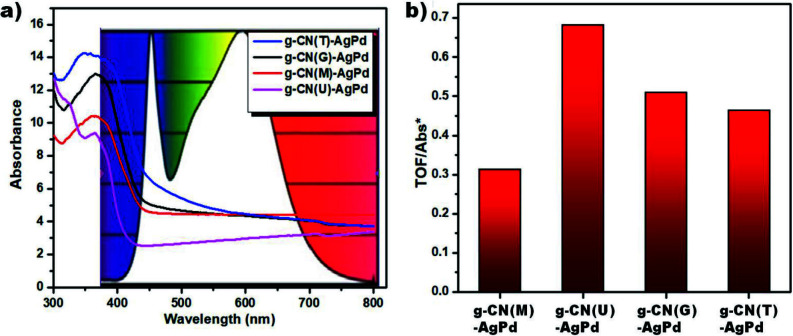
a) Overlapping thes pectrum of the light source and the UV-Vis DRS spectra of the synthesized Schottky junctions. b) Normalized TOF values of the Schottky junctions. (Abs* = the amount of light absorption calculated by the integration of UV-Vis DRS absorption plots between 400 and 700 nm).

In order to reach a valid consequence about which Schottky junction is photocatalytically more active, the differences in light absorption abilities of catalysts should be considered. Figure 8a represents the spectrum of the light source used during photocatalytic activity measurements and the UV-Vis DRS spectra of the synthesized Schottky junctions. It can be inferred that the light absorption ability of g-CN(U)-AgPd is quite lower than others. This may limit the proper evaluation of catalysts in terms of photocatalytic performance. If the light absorption of the catalysts is normalized by dividing the TOF values to the value found by integrating the absorption plots of the catalysts between 400–700 nm, it can be concluded that the highest photocatalytic activity was performed by g-CN(U)-AgPd Schottky junction (Figure 8b).

## 4. Conclusion

The effects of g-CNs synthesized by thermal polycondensation of different precursors on the photocatalytic activity of Schottky junctions were investigated in this report. For this aim, different precursors, urea, thiourea, melamine, and guanidine hydrochloride, were used to synthesize g-CNs, while the photocatalytic dehydrogenation of formic acid was used as a test reaction.

During prolonged polycondensation reaction, different responses to the thermal exfoliation process were detected for g-CNs that are synthesized from the different precursors. These results were associated with the polycrystalline structure of the g-CN which consists of poly (heptazine imide) 4 and triazine-based graphitic carbon nitride 5, while the former can create stronger interactions via hydrogen bonds than the triazine based ones. Thus the strong resistance in g-CN(M) to the thermal exfoliation is attributed to the poly (heptazine imide) richness of the structure. TEM images showed the formation of AgPd NPs of different sizes on different g-CNs, attributed to the different surface morphology of the synthesized g-CNs. It was observed that the smoothest surface (g-CN(M)) has the smallest NPs (2.33 ± 0.51) while the most porous surface (g-CN(U)) has the largest NPs (3.49 ± 1.11 nm). PL, TRPL and XPS analyses revealed that the charge separation ability and charge carrier mobility is the best in g-CN(U)-AgPd which followed by g-CN(T)-AgPd and g-CN(G)-AgPd. However, the photocatalytic activity tests although revealing the high performance of g-CN(T)-AgPd and g-CN(G)-AgPd, the activity of g-CN(U)-AgPd has lagged behind. The low photocatalytic activity of g-CN(U)-AgPd was associated with the poor light absorption ability, while surface defects of g-CN(T)-AgPd make it an attraction center for the substrates, and results in high activity. However, at the end of the day, if the differences in the light absorption capacity of the catalysts are compensated by normalizing the absorption capacity, it can be argued that the most active photocatalyst is g-CN(U)-AgPd.
